# BullVal$: An Integrated Decision-Support Tool for Predicting the Net Present Value of a Dairy Bull Based on Genetic Merit, Semen Production Potential, and Demographic Factors

**DOI:** 10.3390/ani13132062

**Published:** 2023-06-22

**Authors:** Allison Q. Gorr, Victor E. Cabrera, James Meronek, Kent A. Weigel

**Affiliations:** 1 Department of Animal and Dairy Sciences, University of Wisconsin, Madison, WI 53706, USAvcabrera@wisc.edu (V.E.C.); 2 ABS Global Inc., DeForest, WI 53532, USA

**Keywords:** Markov chain, herd simulation, bull valuation

## Abstract

**Simple Summary:**

This study presents a user-friendly model for assessing the value of dairy bulls in artificial insemination (AI) companies. The model, a Markov Chain model, considers important factors for replacement decisions, such as a bull’s age, expected semen production, and predicted genetic merit. Data from a leading AI company were used to apply this model, which calculates a bull’s net present value based on various costs and revenues. The model also calculates the value of a bull compared to a potential young replacement. Findings showed that nearly half of the bulls were recommended for replacement due to a negative value comparison. The value of a bull was primarily influenced by market distribution and pricing, as well as the interaction of semen production with genetic merit.

**Abstract:**

Deciding when to replace dairy bulls presents a complex challenge for artificial insemination (AI) companies. These decisions encompass multiple factors, including a bull’s age, predicted semen production, and estimated genetic merit. This study’s purpose was to provide a practical, objective tool to assist in these decisions. We utilized a Markov Chain model to calculate the economic valuation of dairy bulls, incorporating key factors such as housing costs, collection and marketing expenses, and the bull’s probable tenure in the herd. Data from a leading AI company were used to establish baseline values. The model further compared a bull’s net present value to that of a potential young replacement, establishing a relative valuation (BullVal$). The range of BullVal$ observed spanned from −USD 316,748 to USD 497,710. Interestingly, the model recommended culling for 49% of the bulls based on negative BullVal$. It was found that a bull’s net present value was primarily influenced by market allocation and pricing, coupled with the interaction of semen production and genetic merit. This study offers a robust, data-driven model to guide bull replacement decisions in AI companies. Key determinants of a bull’s valuation included market dynamics, semen production rates, and genetic merit.

## 1. Introduction

Dairy genetics companies seek to provide top genetics from elite bulls to accelerate genetic progress and enhance farm profitability of their customers. Most companies have well-established protocols for acquiring and raising young bulls and collecting, processing, and selling their semen. However, the decision of when to replace a bull with a new selection candidate can be subjective and may be influenced by many factors and their interactions.

Currently, replacement decisions typically involve many individuals, with competing interests, within a given artificial insemination (AI) company, including sire analysts (who acquire the bulls), veterinarians, inventory managers, sales and marketing staff, and barn managers. These individuals must use data regarding genetic potential, semen production, health, temperament, and other factors to decide whether a given bull is likely to: (1) achieve “premium” status in the marketplace and generate millions of dollars in revenue and tens of thousands of offspring; (2) reach (or remain in) “cash cow” status and contribute a modest revenue stream for the foreseeable future, or (3) lag behind its herd mates in revenue-generating potential due to poor semen production and/or a genetic profile that is no longer competitive or marketable. Each bull must be evaluated relative to its existing herd mates at the AI company, as well as potential replacement bulls that may be younger and have higher genetic merit (i.e., opportunity cost), while simultaneously considering the fixed and variable costs associated with keeping the bull or acquiring a replacement.

While objective methods to combine semen production, genetic potential, age, and other factors for valuation of dairy bulls in an AI company context are lacking, such methodologies and decision-support tools are well-established for dairy cows [1,2,3]. These methods combine data regarding age, parity, milk production, pregnancy status, genetic potential, and the inventories of lactating cows and replacement heifers to formulate an estimate of the future income that will be generated by an individual cow, relative to her current and potential herd mates, such that the farmer can decide when to replace a specific cow in an objective and optimal manner (e.g., CowVal$). To our knowledge, this framework has not been extended to the monetization or ranking of dairy bulls based on income-generating potential for an AI company. Focus has been entirely on ranking bulls based on their potential to generate profit for the dairy farmer by producing offspring of greater or lesser genetic potential for individual traits or an overall profit index (e.g., Refs. [4,5])

The most common index used for ranking dairy cattle today is lifetime net merit (NM$), which considers the predicted genetic merit of cows, bulls, heifers, and calves for production, type, health, longevity, fertility, and calving traits relative to an average animal of the same breed [6]. However, the NM$ index does not consider the semen production characteristics of a bull, only the traits he will transmit to his female offspring. As such, NM$ can be considered as an optimal tool by which dairy farmers can rank bulls when purchasing semen, but expected semen production for a specific bull at a given time varies widely [7], and NM$ is not sufficient for making replacement decisions regarding individual bulls based on their likely contributions to the future net profit of an AI company.

Another, possibly most important, defining factor of a bull’s profitability for an AI company is his market appeal. Globally, even within a country, farmers’ needs for genetics and types of bulls differ [8,9,10].

Therefore, the objectives of this study are as follows: (1) provide a user-friendly Markov Chain (MC) model of economic valuation for dairy bulls, focusing on the most important factors contributing to replacement decisions, and (2) describe the features and outcomes of this model when applied to data from a leading AI company.

## 2. Materials and Methods

The replacement problem of a bull was solved by MC as the difference between net present value (NPV) of a bull (NPV bull) and its replacement (NPV replacement), hence:

BullVal$=NPV bull−NPV replacement.

This simple algorithm is the aggregation of a MC model, in which a user can define parameters of a bull and compare with those of a potential replacement, with considerations of age, total sperm production (TSp), and genetic merit.

### 2.1. Markov Chain Bull Model

A dairy bull herd was represented by a 4-month age bin MC model as a matrix. Four months was chosen to model three rounds of replacement decisions per year, based on the current frequency of Council on Dairy Cattle Breeding (CDCB; Bowie, MD) genetic evaluations of US dairy cattle. One state defines potential bull ages: AGE (19 bins of 4-month duration, spanning the period from 10 to 85 months of age).

In a MC simulation, each bull is decomposed through time in all possible states dictated by the transition probabilities, which are then referred to as resulting fractions or proportions of a bull in each iteration. The proportions of a bull represented over time (BULL_AGE_) were simulated through MC following Cabrera (2012) [1]. A vector of transition probabilities represented the probabilities of a bull leaving the herd (CULL) while in a given age bin. The proportion of a given bull explains the probability that a bull in AGE bin i will remain in the herd until the AGE bin i+1. Then, the proportion of a bull remaining in the herd until the next age bin is calculated as:

(BULL_AGE+1_) = (BULL_AGE_)(1−CULL_AGE_);

and a replacement bull enters the herd as AGE = 1, (BULL_1_) as:

$\left({\mathrm{BULL}}_{1}\right)=\overset{19}{\underset{1}{\sum }}{\mathrm{BULL}}_{\mathrm{AGE}}\left({\mathrm{CULL}}_{\mathrm{AGE}}\right)$, which assures that herd size remains constant. 

A bull’s (or replacement’s) probabilistic life was represented from the time the bull entered the analysis (Age Start) until a point in the future when the bull and its potential replacements had reached the MC condition of steady state [1,11,12]. The MC condition of steady state is realized when the proportions of animals in each state no longer change with an increase in time (iteration), regardless of the current state of the bull or its replacement in the first iteration [13].

The model was solved through recursive iterations until the probability distribution of a bull across all states of model reached steady state. In each iteration, aggregated discounted net returns of all probabilities of the bull were estimated for the given 4-month time period. Steady state of the Markov chain was reached after 310 iterations.

To create the MC, assumptions were made to establish a base herd of bulls. Bulls were assumed to enter production at 10 months of age. Once they entered collection status, bulls were collected eight times per month until culled. In practice, bulls removed from collection rarely return; exceptions include injury, illness, or bulls that have not yet reached puberty. In the model, any remaining bulls were culled at age 85 months (7 years 1 month). TSp for a given bull in a 4-month period was treated as a deviation from the mean sperm production for bulls of that age. In a production setting, many factors may impact predicted and actual TSp, including collection frequency, barn personnel, temperament, environmental conditions, semen quality, and processing regimen.

### 2.2. Economic Module

The NPV of a bull or its replacement was the aggregated 4-month discounted (∂) net value over 330 iterations (330 4-month, i) that resulted in NPV bull (value of keeping the bull) or NPV replacement (value of replacing the bull). Economic factors used in this calculation were the incomes and revenues incurred in the maintenance, production, and culling of a bull: (1) income from straw units produced (Si) according to the bulls’ age, predicted TSp deviation, genetic merit, and market; (2) maintenance cost (Mc), including housing, veterinary care, labor, and feed; (3) costs associated with involuntary culling (Cc), including cost of replacement and depreciation, and (4) income generated from involuntary culling a bull (Ci) and salvage value. Therefore,

$$\mathrm{NPV}\;\mathrm{bull}\;\mathrm{or}\;\mathrm{NPV}\;\mathrm{replacement}\;=\sum_{i=1}^{330}\left[\partial \sum_{\mathrm{AGE}=\mathrm{age}}^{\mathrm{AGE}+1}{\left(\mathrm{Si}-\mathrm{Mc}-\mathrm{Cc}+\mathrm{Ci}\right)}_{\mathrm{age}}({\mathrm{BULL}}_{\mathrm{age}})\;\right];$$

where i marked the 4-month time iteration of aggregated NPV calculations, up to 330 iterations. Age defined the bull being analyzed and ranged from 1 to 19. A replacement bull started at AGE = 1, assuming a bull was replaced with a new young sire entering production. A list of minimum variables required to calculate bull value and base values for bull replacement, herd, and economic variables is provided in Table 1.

#### 2.2.1. Bull Variables

*AGE*. Age bin defined age of bull at the starting point (iteration = 1) in the MC model. As explained previously, AGE contained 19 four-month bins, from 10 to 85 months of age.

*Age Class*. To obtain price per unit in the income equation (explained below), age bins were grouped into 3 classes: young (1–4 AGE), in-waiting (5–12 AGE), and proven (12–19 AGE). In-waiting signified a bull that is older than genomic (young) bulls that entered the AI stud recently and younger than proven (old) bulls that already have offspring with performance data. This was done to reduce dimensionality, allow more samples within each grouping, and achieve a more stable price.

*Arrival Age*. Age in months at which a bull arrived at the AI stud. Used to calculate depreciation value within culling cost variable of NPV (depreciation calculation is defined in herd variables section). A bull could arrive at the AI stud at 1 to 15 months of age and might not enter the production herd immediately. Costs associated with rearing bulls prior to production were assumed to be constant across all bulls and are not considered within the NPV calculation.

*Expected TSp percent deviation from mean*. The NPV bull could be calculated assuming a bull’s average TSp production. A bull’s expected production capability, Pdev, was the percent deviation from mean TSp, and was multiplied by the aggregated income generated (Inc) based on average TSp production.

*Net Merit decile bin*. The genetic contribution of a bull was considered using the decile of NM$ for the bull, compared with the herd’s distribution of NM$ values. This value was used to find the price per unit, which was a function of age, market, and genetic merit, in the income calculation.

#### 2.2.2. Economic Variables

*Aggregated discount*. The aggregated discount was defined as: $\partial =\frac{1}{{\left(1+int\right)}^{i+1}}$ where *int* was the interest rate.

*Income from straws produced*. Income generated by unit sales was defined as:

$Si=\left(1+Pdev\right)*\sum_{MKT=mkt}^{6}\left(Inc_{mkt,\;\;NM,age}\right)$ where Pdev: Bull’s sperm production deviation from herd mean, based on the average TSp deviation of a bull for the three most recent trimesters; MKT: Product market (1–6, explained below); Inc: money generated from the sale of product destined to different markets, based on age, TSp production, and NM bin.

*Market pricing and distribution of semen units*. Bulls (and units) were valued differently depending on where the semen was sold. The income equation had the capability to calculate Inc based on the amount of product distributed to each market group (market share).

*TSp per straw unit (packing rate)*. Average number of cells packaged into a unit. The value divided TSp expected per bull into units, which was then multiplied by price per unit to get income from straw units produced.

*Income generated from unit sales*. Money generated from the sale of product destined to different markets, based on age, TSp production, and genetic merit was the product of price per unit (PU), market share percentage (MS), and number of units produced (U):

$$Inc_{mkt,\;NM}=\left(PU_{age\;class,\;NM}\right)*\left(MS_{age,\;NM}\right)*\left(U_{age}\right)$$

*Maintenance cost*. The maintenance cost variable incorporated prices for housing, maintenance, and veterinary costs for a bull.

*Culling cost*. Cost of culling a bull $Cc=CR+CULL_{age}*Depr_{age},\;where$ CR: cost of replacement, which was the purchase price of a new bull; Depr: depreciation cost, which was an aggregated price based on depreciation term and value.

*Depreciation*. Value assigned to a bull for insurance purposes, based on age of bull, arrival age, and term length.

Depreciation cost within the NPV was calculated as Depr = Depreciation Value—(Depreciation Value/(Depreciation term − ArrivalAge) ∗ (age-ArrivalAge)), where Depreciation Value is the original value assessed for a bull, Depreciation term is the length of depreciation realization, and age is the age that is currently being evaluated within the aggregated NPV.

*Depreciation term*. The length for depreciation realization, based on age (month). Depreciation was the remaining cost that must be paid if a bull was culled prior to the depreciation term.

*Culling income*. Income generated from culling the bull, which was simply the product of salvage value (SV, constant across all ages) and proportion of culled animals: $Ci=SV*CULL_{age}.$

*Culling percentage*. Involuntary culling percentage (CULL) per age bin. It was assumed that all bulls were culled at the end of AGE 19.

### 2.3. Case Study

Model performance was demonstrated using production data, sales records, health events, and bull demographics of Holstein bulls at two collection facilities of a commercial AI company (ABS Global Inc., DeForest, WI, USA). Bulls in production from April through November 2020 were used in this illustration (reflective of two trimesters). The MC model for each bull was calculated using age of the bull in April 2020. Genetic predictions for NM$ from the December 2020 CDCB genetic evaluation were used to classify each bull into a decile, and its arrival age was used to calculate depreciation. Each bull’s valuation was relative to a replacement animal of AGE = 1, Arrival age = 6, and NM = 9. It was assumed that a replacement bull would be a young bull at the beginning of its productive life, with the average arrival age, and NM just below that of the most elite bulls used to create the next generation (i.e., NM = 9).

Involuntary Culling Percentage. Involuntary culling percentages, used as transition probabilities, were derived from health records of bull deaths, recommended culling decisions, or actual culls (Table 2).

Expected TSp percent deviation from mean (Pdev; %). This was derived using company collection records from 2018 to 2020, aggregated to an average TSp per age bin, such that each month a bull was collected 8 times (Table 2). For the decision support tool, the user could enter the deviation from this mean. In the herd case study, a bull’s TSp deviation was calculated by averaging deviations from the last 3 (at most) collection months.

**Figure A1:**
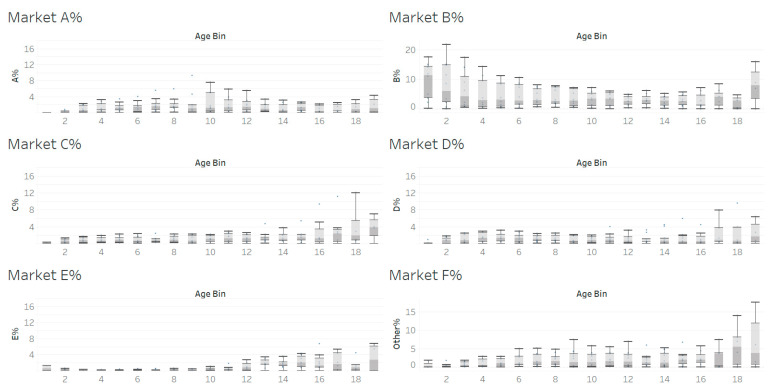
Percentage of product sold to each market A through F across 4-month age bins. Light gray shading indicates upper middle quartile, and dark gray indicates the lower middle quartile.

Market share and pricing. Twenty-nine countries that received more than 200,000 semen units according to 2018 to 2020 sales records were split into 6 market classes. To identify trends in types of products used in each country, PTAs of bulls sold in each country were averaged. The countries were then ranked for each PTA value, providing an estimate of importance that trait has on overall selection by country. Similar countries were grouped together manually. Market A contained 6 countries, in which fertility traits had high importance and milk composition traits had low importance. Market B contained 3 countries, in which milk production traits were of high importance and fertility and type traits had low importance. Market B also contained the domestic market. Market C contained 5 countries, in which milk yield and type composites were of moderately high importance. Market D contained 3 countries with high importance for type composites. Market E contained 6 countries, in which PTA Milk, Productive Life, SCS, and Net Merit were of high importance. Lastly, Market F contained 6 countries that did not fit into above groupings and lacked a discernable pattern in traits of high importance. Once grouped, average price per group was calculated per age bin. A smoothing function was applied to each market to limit the influence of outliers (see below). Market share was calculated as the percentage of total units per age group directed to each market. Figure A1 in Appendix A contains box plots of market percentages across age bins. Table A1 in Appendix A contains prices by NM bin, market, and age class.

Unit price smoothing function. Price per unit of semen was estimated based on age class (young, in-waiting and proven), market class, and NM$ decile (Table A1 in Appendix A). Age class was used to decrease dimensionality of ages, while capturing price differences between young, in-waiting, and proven bulls. Sales data obtained from the company did not include the actual price received for each unit of each bull in a specific country. Rather, a blended price was available for all bulls sold to the country in that transaction, which reflected the average price per unit across all bulls in the order; this tended to dilute variation in prices per unit of different bulls, especially when high-value and low-value bulls were grouped in the same order. Sales records from 2018 to 2020 were filtered to remove outliers. Bulls were classified into age class, market, and NM$ bin groupings at the time of sale. Empirical Bayes was used to smooth the price estimates (Martin, 2018). The empirical Bayes method provides a balance between group estimates and the population mean, such that population mean carries more weight for groups with limited information. In this case, prices within age class and NM decile were blended with population means for prices in a given market, as shown below:

$$\beta_{i}=\frac{\tau^{2}}{\tau^{2}+\varepsilon_{i}^{2}}$$

where

β_i_ = interpolation factor

$\tau^{2}$ = population variance

$\varepsilon_{i}^{2}$ = standard error in the price of group i, which is σ^2^_i_/n_i_

$$\mathrm{shrunk}\;\mathrm{price}\;={\;\beta }_{i}{\bar{x}}_{i}+\left(1-\beta_{i}\right)\mu$$

µ = population mean

${\bar{x}}_{i}$ = average price per group *i*

Maintenance cost. The AI company used in this case study assigns an estimate of USD 30/bull/day for covering all physical maintenance costs, such as feeding, housing, and veterinary costs. This value was adjusted to fit 4-month age bin and remains the same across all bulls in the herd.

Depreciation value and term length. The company insured bulls for USD 54,000 for 36 months (depreciation term).

## 3. Results and Discussion

### 3.1. Performance of the Model and Results of Base Scenario at Steady State

The base scenario was derived from the case study, subsequently all results pertain to the case study. Across 330 iterations, steady state was reached around the 310th iteration, based on BULL_AGE = 1_. The SD between the 310th and 330th iterations for AGE = 1 was 0.137%, showing that there was minimal variability in the proportions between iterations. The replacement bull (AGE = 1, NM Bin = 9, Arrival Age = 6) had a discounted NPV of USD 250,951. This was broken down into maintenance cost of USD 63,600, culling cost of USD 7264, income from culling of USD 532, and income from semen sales of USD 321,283. Adjusting any input values of a replacement bull would change his NPV and the BullVal$ of the herd, but it would not change the overall ranking of bulls within the herd.

Market prices (Table A1 in Appendix A) were established using an empirical Bayes smoothing function. Contrary to intuitive thinking, the NM Bin 10 reflected average market price. We speculate that this may be due to pairing of elite bulls with lower-demand bulls as “blend” packages, where fewer units from elite bulls are sold with greater numbers of units from inexpensive bulls that are more readily available; this would reduce the valuation of genetically elite bulls in our case study analysis. We chose to use semen prices derived in this manner for the case study, despite the aforementioned limitations in data clarity, but future users may have access to more precise pricing data at the individual bull level for specific markets.

### 3.2. Case Study

**Figure A2:**
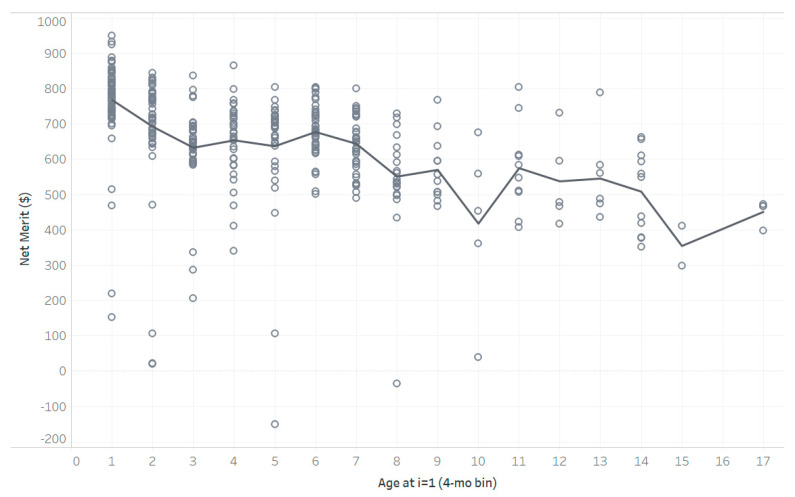
Raw NM$ values of case study bulls by age bin they were in April 2020. Open circles indicate individual bulls, and the line indicates mean NM$ by age bin.

A total of 396 Holstein bulls collected from April to November 2020 made up the herd. Table 3 shows the distribution of bulls in each NM bin, with an average of 7.9. With the knowledge that the deciles were established using bulls collected from 2018 to 2020, the company’s herd had a higher NM than previous trimesters or years, a trend that was expected. The genetic trend can be observed in Figure A2 in Appendix A, a plot of the herd bulls’ raw NM$ with their ages in April 2020. As age decreased, NM$ increased, showing that younger bulls had higher NM, except those that were chosen for specialty markets, like high genetic merit for type conformation.

**Figure 1:**
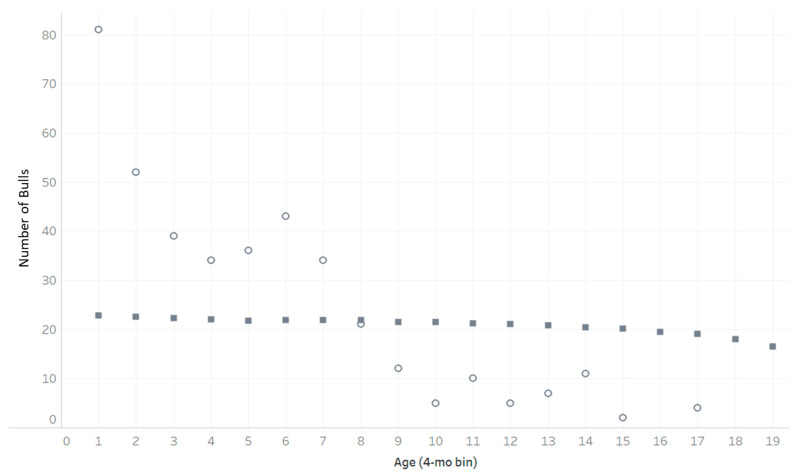
Distribution of herd demonstration bulls across the AGE bins in which they start the MC model in (i = 0, open circles) and at steady state (i = 310, closed squares).

The average arrival age was 6 month, and average age bin of these bulls at the start of the MC was 5, ranging from 81 in AGE = 1 and 2 in AGE = 15 at *i* = 1 (Figure 1). The herd distribution by age at steady state ranged from 16 bulls in AGE = 19 to 23 bulls in AGE = 1. The drastic difference in herd distribution between *i*= 1 and *i* = 310 demonstrates decisions that cannot be captured due to data limitations.

**Figure 2:**
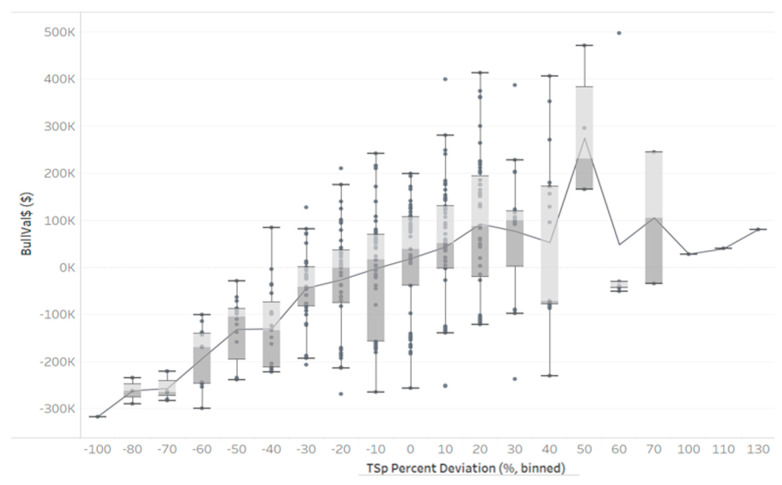
Boxplot of herd bulls’ total sperm (TSp) deviation from mean (%) and their bull valuation (BullVal$). Light gray shading indicates upper middle quartile, and dark gray indicates the lower middle quartile.

The percentage of product sold to each market by age is portrayed in Figure A1 in Appendix A. Market B dominated the market share in young bulls, whereas other markets increased their share as bulls aged. International sales relied more heavily on older, proven bulls. The herd’s BullVal$ ranged from −USD 316,748 to USD 497,710. Deviations from mean TSp ranged from −94% to 139% (Figure 2).

For TSp deviation bins with more than one observation, wide ranges of BullVal$ were realized. As expected, with an increase in TSp, the overall trend of BullVal$ increased. Bulls with high BullVal$ did not have the highest TSp, but most tended to be above the mean.

A previous study showed that TSp forecasts up to 4 months into the future were reliable [7]. It would be feasible for a company to incorporate TSp forecasts as opposed to deviations from mean TSp, but this would not drastically change the BullVal$ ranking.

**Figure 3:**
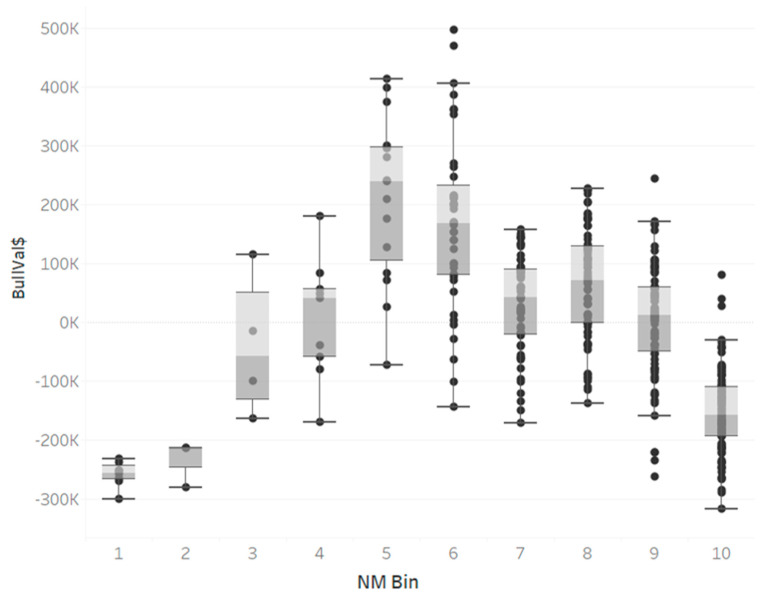
Boxplot of herd’s bull valuations across different net merit (NM) decile bins. Light gray shading indicates upper middle quartile, and dark gray indicates the lower middle quartile.

To explore the relationship between NM$ and BullVal$, Figure 3 plots NM bin with BullVal$. The expected relationship between BullVal$ increasing with NM bin was not observed across all bins.

In the first 5 NM bins, there was an increase in value, with the lowest BullVal$ in lowest NM bins. However, there was a decrease in average BullVal$ from NM bin 6 to 10. A possible explanation for this decrease is that higher NM bins have younger bulls, with Age Start mean of 2.26 for NM bin 10; very little production data were available for these bulls, so a TSp deviation might not be an accurate portrait of the bull’s lifetime potential. For NM bin 10, TSp deviation was 3.55 ± 41.58% and NM bins 7 and 8 had TSp deviations below mean (Table 3). Young bulls beginning the production process have varying performance, as they are new to the collection process and have yet to reach maturity. Other possible reasons why the average BullVal$ was lower than expected for higher NM$ bulls are reservations of elite bulls for contract matings, or package deals where high value bulls’ units are sold in limited quantities with large quantities of lower NM$ bulls’ units. The first example highlights rare cases which elite bulls’ semen may not be immediately available for sale, or if a sale is allowed, a contract is bound to the offspring, which would skew the price of units. The latter, more probable, reason would lead to skewed blended prices within sales records, driving down the apparent market price for elite bulls. The sales data provided assigned a blended price across the whole order, so the high-valued units were recorded at a lower price, heavily influenced by the mass lower-priced units. To establish market prices, empirical Bayes smoothing function was used in attempt to smooth outliers and blended prices. With so few records of elite bull unit sales, the smoothing function set the market prices to average, which decreased elite bulls’ values. If actual bull-level sales data were attainable, one would expect a bull of higher NM$ to have a higher BullVal$, as long as his TSp was above average. TSp and NM$ contribute to BullVal$, but there are also other intangible factors, such as market distribution and pricing, that contribute to a bull’s potential net revenue.

**Figure 4:**
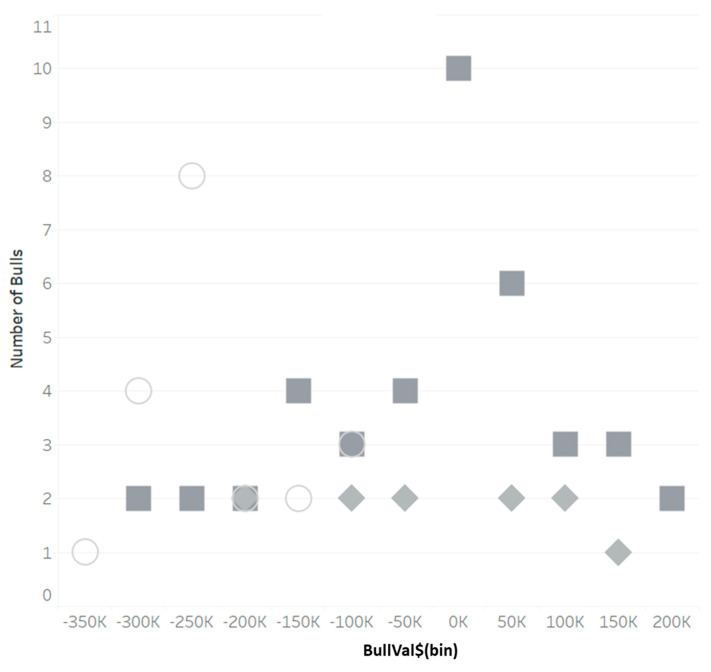
Histogram of bulls added (open circle), involuntarily culled (closed square), and voluntarily culled (closed diamond) from April 2020 through November 2020 in herd demonstration with their assigned bull valuations (BullVal$).

BullVal$ would be a beneficial tool in culling decisions as well as determining early on if a bull would be worth adding to the herd (if his predicted TSp and NM$ would jointly be beneficial in a profitable market). Figure 4 shows the number of bulls per each USD 50,000 BullVal$ bin added, involuntarily culled, and voluntarily culled between the August and December 2020 trimesters. Logistically, we would like to see bulls added to the herd with positive BullVal$ bins and conversely, culled bulls with negative BullVal$; however, this did not hold true with the case study herd. Out of the 20 new bulls added to the collection herd, all bulls had negative BullVal$. Out of 41 voluntary-culled bulls, 17 (41%) bulls had a BullVal$ below USD 0.

This model and case study had limitations and challenges. First, the sales data available for this study were average sales prices for orders, which could contain multiple bulls, all averaging to the same price. This does not accurately portray the actual sales price of a bull semen. Moreover, a company may sacrifice on sales price to foster a budding relationship with a new market, undervaluing bulls and losing present revenue for (hopeful) future gain. Additional business relationships, contracts, and government regulations, among other constraints are not considered in this study, but would play significant roles in pricing and market distribution. Lastly, the adoption of an objective tool can be a challenge when competing interests exist, and greater insight into the performance of this bull valuation tool may have been gained if the authors had access to data from several commercial AI companies. It would be beneficial for the tool to be modified or updated to reflect market changes and bull herd demographics of specific companies. Future studies should consider differences in reliability between young, genome-tested bulls and older bulls with milking daughters, because this uncertainty could cause some bulls to change NM bins.

The authors suggest that this tool would be most beneficial in culling decisions when tied into the product allocation and collection scheduling process. Bulls with negative BullVal$ should be culled before high BullVal$ bulls (barring any health issues), to make way for more profitable replacements. An example of how this may fit into a collection scheduling process is in a situation where collection spots are limited, we would prioritize higher BullVal$ bulls for those spots. A similar case can be made with product allocation: assigning higher BullVal$ bulls to markets would capitalize on the potential net revenue. Again, this model would need to be updated routinely (2–3 times/year) to reflect the current bull population and market characteristics.

## 4. Conclusions

The present study demonstrated that a Markov chain model can be used to provide economic valuation of dairy bulls, while focusing on the most important factors contributing to replacement decisions, such as age, predicted semen production, and predicted genetic merit. The Markov chain model allows for user-defined input based on current replacement policy and bull demographics. This model provides a new metric of ranking and valuing bulls based on their actual contribution to revenue of the company. A negative bull value indicates that the chosen bull is less profitable than the predicted discounted lifetime profit of a new young sire of average production capabilities, suggesting that the bull should be culled. A case study demonstrated the tool’s feasibility of valuing and ranking a herd and highlighted pitfalls with data availability. The range of BullVal$ encountered was −USD 316,748 to USD 497,710, with 49% of bulls recommended for culling based on negative BullVal. A bull’s NPV was influenced primarily by market allocation and pricing, as well as the interaction of sperm production with genetic merit. This decision support tool is contained within an Excel workbook, allowing individual bull valuation and whole-herd assessment.

## Figures and Tables

**Table 1:** Variables required to calculate bull value using the MC model, and base values used in model illustration.

■ Variable	Base Value
■ Bull variable evaluated	
■ Current age bin	Bull-specific (1 to 19)
■ Replacement bull variable	
■ Age bin	1
■ Net Merit bin	9
■ TSp deviation	0
■ Arrival age (month)	6
■ Herd variable	
■ Bulls in herd	396
■ Economic variable	
■ Maintenance cost (USD/bull/month)	900
■ Depreciation cost (USD)	54,000
■ Depreciation term (month)	36
■ Cost of replacement (USD)	10,000
■ Salvage value (USD)	850
■ Packing rate (TSp/straw unit)	15,000,000
■ Interest rate (%/year)	6.00
■ Market price	See Figure A1 in Appendix A for distribution and Table A1 in Appendix A for price (Appendix A)

**Table 2:** Average Total Sperm (TSp) production and involuntary culling proportions across the 4-month age bins generated using production records from 2018 to 2020. It is assumed all animals are culled after AGE 19.

Age (Month)	AGE (4 Month)	TSpBin Ave (Billion Cells)	Involuntary Culling Proportion (%)
10 to 13	1	99.6	0.0067
14 to 17	2	229.3	0.0040
18 to 21	3	296.0	0.0014
22 to 25	4	342.9	0.0041
26 to 29	5	366.3	0.0030
30 to 33	6	386.7	0.0063
34 to 37	7	403.8	0.0104
38 to 41	8	399.8	0.0162
42 to 45	9	415.2	0.0100
46 to 49	10	398.1	0.0148
50 to 53	11	406.5	0.0088
54 to 57	12	422.8	0.0215
58 to 61	13	450.6	0.0202
62 to 65	14	489.2	0.0094
66 to 69	15	485.6	0.0250
70 to 73	16	486.0	0.0200
74 to 77	17	469.2	0.0571
78 to 81	18	433.7	0.0789
82 to 85	19	259.4	1.0000

**Table 3:** Lifetime Net Merit (NM$), number of bulls, mean TSp deviation % (and standard deviation; SD), and mean bull valuation (BullVal$) and SD NM decile bin for the modeled herd.

NM Decile Bin	NM$ Range	Number of Bulls	TSp Mean Deviation % (SD)	Mean BullVal$ (SD)
1	−151 to 152	8	4.2 (31.7)	−257,759 (21,280)
2	205 to 286	3	−29.9 (26.9)	−235,546 (38,630)
3	298 to 351	4	−3.5 (33.7)	−40,291 (120,230)
4	361 to 418	9	5.0 (32.5)	7151 (103,780)
5	423 to 474	15	−0.3 (26.8)	210,924 (142,140)
6	479 to 559	40	1.7 (29.3)	16,8307 (147,720)
7	560 to 636	55	−3.0 (21.0)	30,979 (83,700)
8	637 to 692	76	−6.9 (22.4)	64,302 (93,650)
9	693 to 756	87	4.8 (28.8)	3953 (94,440)
10	757 to 950	99	3.6 (41.6)	−151,324 (−71,390)
Total		396	0.3 (30.9)	−2565 (148,450)

## Data Availability

Data are unavailable due to privacy restrictions of the company who provided the data for the research.

## Appendix A

**Table A1:** Prices assigned to each market based on age class and NM Bin using empirical Bayes smoothing function.

		Market Price (USD/Dose)					
Age Class	NM Bin	A	B	C	D	E	F
Young	1	8.96	7.10	14.65	7.10	7.10	9.81
In-waiting		7.39	7.08	8.94	15.29	6.87	8.48
Proven		4.18	6.04	7.07	5.54	5.00	5.74
Young	2	8.84	7.10	14.81	7.10	7.10	9.43
In-waiting		7.45	7.30	8.02	12.74	3.52	6.52
Proven		3.81	6.52	5.35	5.94	3.82	6.69
Young	3	7.60	5.00	9.10	7.10	7.10	9.39
In-waiting		9.08	4.47	6.71	8.92	4.93	5.12
Proven		6.89	5.66	6.33	15.67	2.31	5.25
Young	4	7.89	4.15	11.52	7.10	9.52	7.24
In-waiting		5.90	6.21	6.67	9.97	2.40	6.81
Proven		9.03	6.24	7.84	7.10	3.38	3.48
Young	5	7.09	4.50	9.19	7.10	5.68	9.59
In-waiting		9.24	5.03	5.56	7.10	3.21	5.03
Proven		7.10	8.46	5.98	7.10	9.06	3.73
Young	6	9.19	6.37	10.51	7.10	8.49	9.62
In-waiting		9.06	9.04	7.74	10.31	7.37	6.66
Proven		4.67	7.19	3.10	7.10	7.10	4.44
Young	7	8.99	6.72	5.14	9.34	8.83	7.74
In-waiting		8.31	7.16	5.99	7.10	7.10	6.48
Proven		7.10	7.10	3.59	7.10	7.10	4.30
Young	8	8.43	5.91	7.97	8.23	14.01	7.87
In-waiting		8.71	7.50	2.82	9.30	7.10	3.71
Proven		7.10	7.10	7.62	7.10	7.10	3.41
Young	9	9.53	5.16	8.02	8.15	7.10	9.41
In-waiting		9.38	7.00	4.96	7.10	7.10	6.56
Proven		7.10	7.10	7.10	7.10	7.10	7.10
Young	10	8.55	5.64	12.37	9.47	7.10	8.43
In-waiting		8.26	7.00	8.21	10.30	7.10	8.70
Proven		7.10	7.10	7.10	7.10	7.10	7.10
